# Supplementary material for: The Regular Consumption of Nuts Is Associated with a Lower Prevalence of Abdominal Obesity and Metabolic Syndrome in Older People from the North of Spain
Source: Int J Environ Res Public Health. 2022 Jan 23;19(3):1256. doi: 10.3390/ijerph19031256 (PMC8834977; doi:10.3390/ijerph19031256)
Supplement: Supplementary file 1 [file ijerph-19-01256-s001.zip › ijerph-1492616-supplementary.pdf]

Supplementary materials

Table S1. Prevalence of BMI, metabolic syndrome and its components by gender.

	Men (N=103)	Women (N=161)	p-value[1]	Total (N=264)
	n (%)	n (%)		n (%)
**Metabolic syndrome**	49 (47.6)	57 (35.4)	0.049	106 (40.2)
**Abdominal obesity**	80 (77.7)	128 (79.5)	0.722	208 (78.8)
**Hypertension**	89 (86.4)	121 (75.2)	0.027	210 (79.5)
**Hiperglycaemia**	47 (45.6)	37 (23.0)	<0.001	84 (31.8)
**Hypertriglyceridemia**	21 (20,4)	38 (23.6)	0.541	59 (22.3)
**Low HDL-c**	21 (20.4)	31 (19.3)	0.821	52 (19.7)
**Normal weight**	13 (12.6)	51 (31.7)	<0.001	64 (24.2)
**Overweight**	62 (60.2)	70 (43.5)	0.008	132 (50.0)
**Obesity**	28 (27.2)	40 (24.8)	0.664	68 (25.8)

[1]Differences between gender were evaluated using the Pearson Chi square Goodness-of-Fit test.

Table S2. Percentage of number of variables diagnosing metabolic syndrome (hypertension, hyperglycaemia, hypertriglyceridemia and low HDL-c) by gender.

Number of variables MetS	Men (N=103)	Women (N=161)	p-value[1]	Total (N=264)
	n (%)	n (%)		n (%)
**none**	6 (5.8)	32 (19.9)	0.002	38 (14.4)
**1**	28 (27.2)	74 (46.0)	0.002	102 (38.6)
**2**	37 (35.9)	36 (22.4)	0.017	73 (27.6)
**3**	23 (22.3)	17 (10.6)	<0.009	40 (15.2)
**4**	9 (8.7)	2 (1.2)	0.010	11 (4.2)

[1]Differences between gender were evaluated using the Pearson Chi square Goodness-of-Fit test
